# Applications of lump, breather, rogue, and various nonlinear waves in the modeling of blood vessels: analysis of the nonlinear Murray equation with heterogeneous wall properties

**DOI:** 10.1007/s11571-026-10513-4

**Published:** 2026-07-28

**Authors:** Sajawal Abbas Baloch, Aysha Zafar

**Affiliations:** 1 Department of Mathematical Sciences, University of Stellenbosch, Stellenbosch, South Africa; 2 Department of Mathematics, The Islamia University of Bahawalpur, Bahawalpur, Pakistan

**Keywords:** Soliton solutions, Bilinearization, Periodic waves, Ansatz functions, Nonlinear waves

## Abstract

In order to gain a deeper understanding of nonlinear wave dynamics, soliton solutions play an important role. In this research, using suitable transformations, we rigorously examine a wide range of wave solutions for the nonlinear Murray equation, exhibiting new wave patterns, including lump, lump with one kink, lump with two kink, rogue waves, periodic, breathers and multi-waves solutions. In individuals with cardiovascular illness, these solutions can be utilized to increase blood flow since they reflect a consistent fluctuation in blood vessel shape and diameter. Under the pathological conditions, localized pressure pulse occur in arteries, for which lump type solutions can be used as mathematical analogues. Rogue waves can describe sudden extreme spikes in blood pressure. Moreover, multi-wave solutions play in important role in hemodynamics, particularly in modeling the nonlinear arterial systems. These solutions have recently emerged in the literature, providing researchers with a crucial tool for understanding intricate biological systems. To illustrate the characteristics and development of the solutions, comprehensive graphical representations are provided, including 3D surface plots, 2D profiles, and contour plots. By examining these plots, we analyze how parameters affect the amplitude and propagation behavior of nonlinear waves which are related to change in the geometry of blood vessels. The derived solutions can deepen our comprehension of complex biological systems and may help in the development of new medical models and treatments. To the best of our knowledge, this is the first study to report such a diverse class of exact wave solutions for the nonlinear Murray equation with heterogeneous wall properties.

## Introduction

Nonlinear partial differential equations (NLPDEs) have been widely used to describe nonlinear processes in many complex areas of natural sciences, particularly in mathematical physics, biology, and engineering (Xiang et al. 2024; Han et al. 2025; Zhao et al. 2026; Ullah et al. 2024). In the many scientific and technical domains, the numerous significant natural problems are expressed mathematically by the NLPDEs. For the purpose of creating, examining, and resolving mathematical models, the precise wave solutions of NLEEs are crucial. The wave solutions for NLEEs, which make a major contribution in nonlinear mathematical physics, are a subject of great interest to scholars. Consequently, the wave solutions can elucidate and develop numerous problems in natural phenomena, such as wave propagations, solitary waves, vibration systems, and thermal systems. We can gain a better understanding of these phenomena by studying the exact solutions to these waves, which serve as mathematical models of the phenomena. In order to comprehend the tool of the physical models (Islam et al. 2025; Khan et al. 2025), the exact solutions of NLEEs may offer a wealth of tangible information. The characteristics of differential equations can also be learned from the exact solutions of NLEEs. Thus, using Wolfram Mathematica and other programming languages to obtain exact solutions for NLEEs has garnered a lot of attention.

Many effective techniques have been offered by a wide range of physicists and mathematicians such as improved modified simplest equation approach (Al-Amin et al. 2025), $\left( {\frac{{G'}}{{{G^2}}}} \right)$-expansion approach (Ullah et al. 2025; Ur Rahman et al. 2026; Usman and Ullah 2025), unified method (Rahaman et al. 2025; Ullah et al. 2023), Riccati Bernoulli sub-ODE approach (Rahman 2026; Hassan and Abdelrahman 2019), modified F-expansion scheme (Iqbal et al. 2025; Apriliani and Maulidi 2020), sine-cosine scheme (Moghaddam et al. 2009; Mirzazadeh et al. 2015), tanh-coth approach (Wazwaz 2008; Parkes 2010), generalized Kudryashov method (Gepreel et al. 2017; Akbar et al. 2021), generalized Riccati equation scheme (Zayed and Alurrfi 2015; Akram et al. 2021), Kudryshov approach (Aldwoah et al. 2025), tanh-sech method(Guo et al. 2019), modified direct algebraic approach (Ullah et al. 2024; Bilal et al. 2025; Ullah 2024; Bilal et al. 2025a, b), the advanced auxiliary equation technique (Akram et al. 2023; Al-Amin et al. 2025), the bilinear neural network approach (Akram et al. 2026), generalized exponential rational function approach (Al-Amin 2025), Hirota bilinear approach (Akram and Ahmad 2024; Ullah 2023), test function approach (Chen and Lü 2022; Evans 1989), $\left( {\frac{{G'}}{G}} \right)$- expansion method (Ullah et al. 2024), Jacobi elliptic function approach (Ullah and Akter 2025) and many more (Iqbal et al. 2025; Baloch et al. 2025; Iqbal et al. 2025; Ullah et al. 2024).

Recent advances in biomedical sciences have significantly enhanced the understanding of vascular structure, metabolic regulation, and endothelial interactions, thereby providing deeper insights into blood flow dynamics and related physiological processes (Wen et al. 2025; Lin et al. 2025; Kang et al. 2025). In biomechanics, research on the nonlinear Murray equation (NLME) and its extensions is still very important. The progress of innovative treatments for a variety of cardiovascular disorders and other conditions affecting blood circulation can greatly benefit from an understanding of the mathematical underpinnings of blood vessel construction and function (Lindström et al. 2015). By addressing the mathematical modeling gaps present in these systems, the investigation into the NLME holds the potential to improve theoretical comprehension and practical applications within the medical sciences, particularly in the advancement of therapies for conditions related to blood circulation (Alzhanov et al. 2024; Owen et al. 2018). Research on the NLME has the potential to improve theoretical comprehension and practical applications in the medical sciences, particularly in the progress of medications for diseases related to blood circulation.

The Murray principle, or Murray’s law, is a physical law that describes how the size of a blood vessel affects how fast blood flows through it. It combines ideas from fluid dynamics and the shape of the blood vessel, as well as other relevant considerations. It can be mathematically represented by the nonlinear Murray equation (Inc et al. 2024).

1.1 $$\begin{aligned} {R^3} = c\left( {\frac{{{G_b}}}{{{\lambda _b}}}} \right) + d\left( {\frac{{{\lambda _b}}}{{{k_\nu }}}} \right) , \end{aligned}$$

where *R* is the blood vessel’s radius, $G_b$ is the rate of blood flow, $\lambda _b$ is the blood’s viscosity, $k_\nu$ is the vessel’s length, and *c* and *d* are included as constants. The analysis centers on the subsequent form of equation.

1.2 $$\begin{aligned} {u_t} = D(u){u_{xx}} + H(u){u_x} + T(u), \end{aligned}$$

where *u*(*x*, *t*) is an unknown function while *D*, *H* and *T* are arbitrary smooth function dependent on *u*. When *D = 1*, $H = {c_1},$ and $T(u) = {c_2}u - {c_3}{u^2}$, where ${c_i}(i = 1,2,3)\in \mathbb {R}$, the Eq. (1.2) will be

1.3 $$\begin{aligned} {u_t} = {u_{xx}} + {c_1}u{u_x} + {c_2}u - {c_3}{u^2}, \end{aligned}$$

which is the a more sophisticated form of NLME.

Numerous researchers have used a variety of techniques to examine the NLME. In Inc et al. (2024), Inc et al. investigated the same model and obtained solitary wave solutions. Mateen et al. (2025) investigated Eq. (1.3) using new auxiliary equation approach and derived soliton solutions. Muhammad et al. (2025) studied fractional NLME using generalized approach and derived several soliton solutions. In past research (Inc et al. 2024; Mateen et al. 2025; Muhammad et al. 2025), most of the efforts were centered around finding classical soliton solutions through analytical techniques. Most of these studies have concentrated on the simplistic modeling of the geometrical structure and homogeneous nature of the walls of blood vessels. In some current research efforts, the Murray equation is generalized into its fractional form, and limited types of solutions, usually solitary or periodic wave shapes, are found. However, the nonlinear wave interaction between different waves for instance, lump-kink or lump-periodic interactions is still unexplored.

**Fig. 1:**
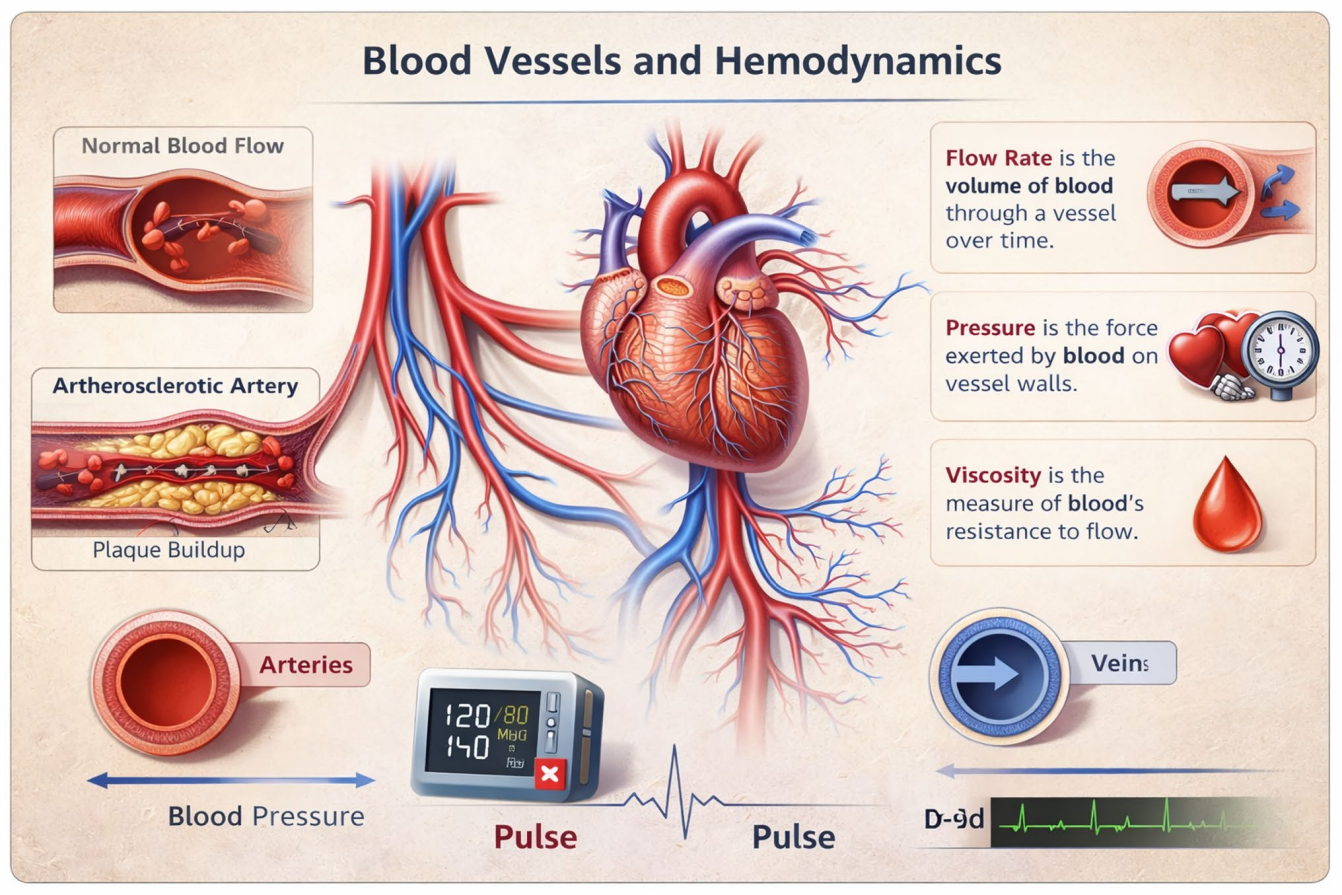
Representation of blood vessels and hemodynamic behavior, illustrating the propagation and interaction of nonlinear pressure waves in arterial systems

The analysis of wave solutions in the NLME is motivated by their crucial role in modeling the blood flow dynamics, specifically for cardio vascular diseases. Previous studies have not provided rigorous analytical solutions which can be used to describe the complex wave dynamics in biological systems. This article closes this gap by constructing new wave solutions with the help of sophisticated mathematical methods yielding deeper understanding on hemodynamics at the same time pushing forward nonlinear wave theory in biomathematics. In this work,using ansatz transformations, we derived lump solutions (LS), lump with one kink $(L_1K)$, lump with two kink $(L_2K)$, roge waves (RWs), breathers waves (BWs), periodic waves (PWs), and multi-waves (MWs) solutions. During the last decade, several techniques have been developed to find exact solutions of NLEEs. Auxiliary equation approach is among one of the most powerful and useful tools that can be applied to find out different kinds of exact solutions of NLEEs based on solvable ordinary differential equations. But, this methodology suffers from some disadvantages and cannot address complex interaction cases. On the other hand, the F-expansion method, which leads to different kinds of periodic and solitary wave solutions using polynomials, becomes less useful while handling highly nonlinear structures and multiwave interaction cases. In this respect, the approach discussed here in this article proves to be more flexible in terms of solving nonlinear equations, allowing us to find out a variety of wave structures such as lump wave, rogue wave, breather solutions, along with their interaction solutions.

The article is arranged as: The LS is covered in Sect. “Lump solution”. $L_1K$ will be covered in Sect. “Lump with one kink solution”, whereas $L_2K$ solutions will be given in Sect. “Lump with two kink solution”. Section “Rogue waves solutions will address the RWs solution. BWs solution is covered in Sect. “Breathers wave solutions”, while PWs solution is discussed in Sect. “Periodic wave Solution”. In Sect. “Multi-wave solution”, we provided MWs solutions. Results and discussion of the solutions are discussed in Sect. “Results and discussion”. In Sect. “Conclusions”, we summarize our results.

**Fig. 2:**
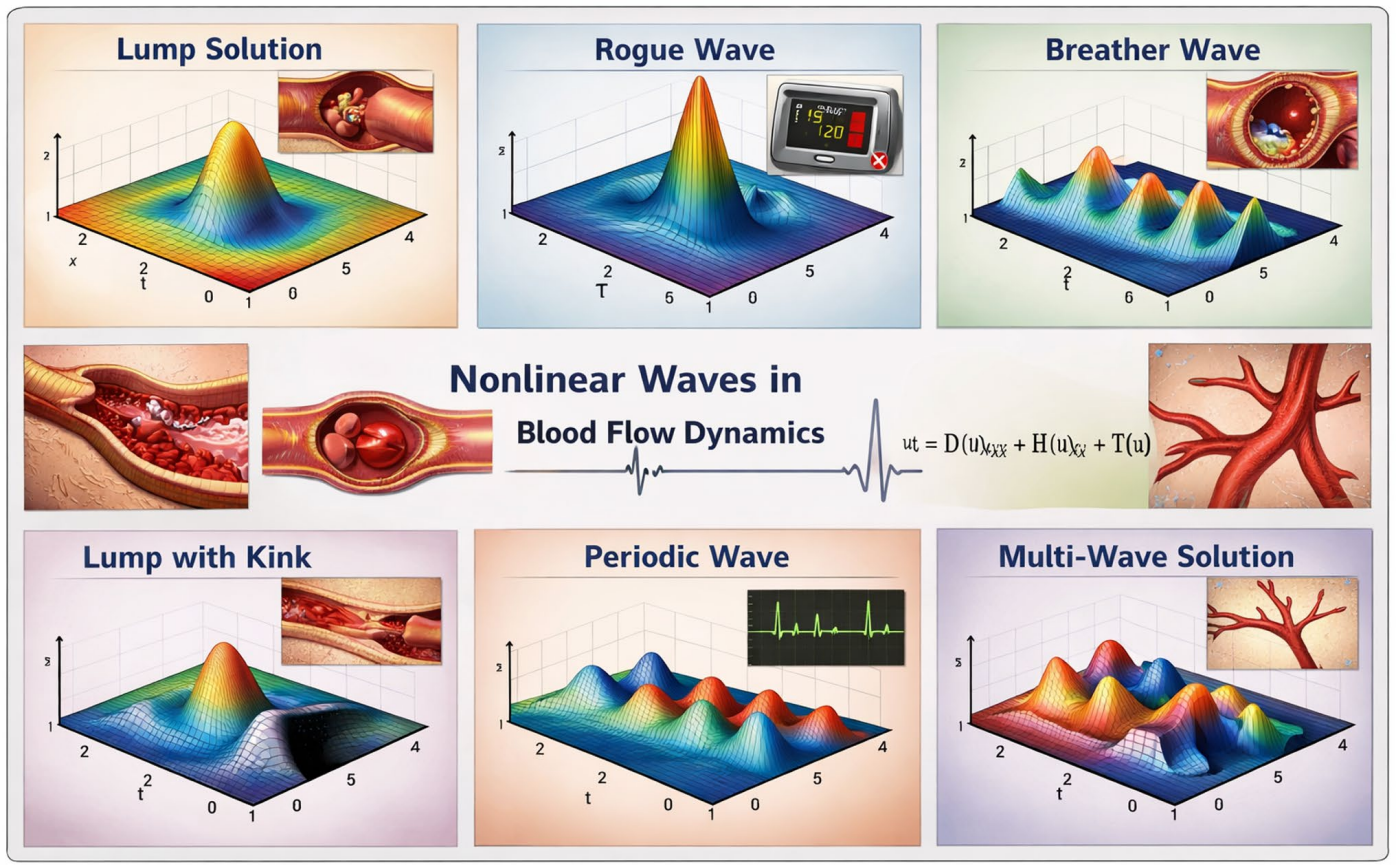
Graphical representation of lump, rogue, breather, periodic, and multi-wave structures and their qualitative interpretation in arterial blood flow

## Lump solution

To determine the wave solution, use the subsequent transformation (Rizvi et al. 2025).

2.1 $$\begin{aligned} u = 2{(\ln f)_x}, \end{aligned}$$

The bilinear form presented below can be obtained by simply substituting Eq. (2.1) into Eq. (1.3). We will derive required derivatives as follows:

$$\begin{aligned} {u_t} = 2\left( {\frac{{{f_{xt}}f - {f_x}{f_t}}}{{{f^2}}}} \right) , \quad {u_x} = 2\left( {\frac{{{f_{xx}}f - f_x^2}}{{{f^2}}}} \right) \end{aligned}$$,

and

$$\begin{aligned} u_{xx} = 2\left( \frac{f_{xxx}}{f} - 3\frac{f_x f_{xx}}{f^2} + 2\frac{f_x^3}{f^3}\right) . \end{aligned}$$

By replacing these derivatives in Eq. (1.3) and simplifying, we obtain the subsequent form:

2.2 $$\begin{aligned} & {c_2}{f^2}{f_x} + f{f_t}{f_x} - 2{c_3}ff_x^2 + 2f_x^3 - 2{c_1}f_x^3 \nonumber \\ & - {f^2}{f_{xt}} - 3f{f_x}{f_{xx}} + 2{c_1}f{f_x}{f_{xx}} + {f^2}{f_{xxx}} = 0. \end{aligned}$$

For LS, we examine the function *f* as follows:

2.3 $$\begin{aligned} f = \eta _1^2 + \eta _2^2 + {j_7}, \end{aligned}$$

where ${\eta _1} = {j_1}x + {j_2}t + {j_3},$ ${\eta _2} = {j_4}x + {j_5}t + {j_6},$ and ${j_i}(0 \le i \le 7)$ are constants. By replacing Eq. (2.3) into Eq. (2.2), we obtain several equations that provide parameter values by reducing all of the coefficients of the *x*, and *t* to zero, as

**Case 1:**

2.4 $$\begin{aligned} {\left\{ \begin{array}{ll} {j_1} = 0,\\ {j_3} = - \frac{{j_4^2\left( {2j_2^2{c_1} - 6j_5^{{\hspace{1.0pt}} 2}{c_1} - 3j_2^2 + 3j_5^2} \right) }}{{{j_2}\left( {j_2^2 + j_5^{{\hspace{1.0pt}} 2}} \right) }},\\ {j_6} = 0,\\ {j_7} = - \frac{{3j_4^4\left( {2j_2^2{c_1} - 6j_5^{{\hspace{1.0pt}} 2}{c_1} - 3j_2^{{\hspace{1.0pt}} 2} + 3j_5^2} \right) \left( {6j_2^{{\hspace{1.0pt}} 2}{c_1} - 2j_5^2{c_1} - 5j_2^2 + j_5^2} \right) }}{{j_2^2{{\left( {j_2^2 + j_5^2} \right) }^2}}},\\ {c_2} = 0,\\ {c_3} = \frac{{j_2^2 + j_5^{{\hspace{1.0pt}} 2}}}{{4{j_4}{j_5}}}. \end{array}\right. } \end{aligned}$$

To obtain the LS of Eq. (1.3), replace Eq. (2.4) into Eq. (2.3) and apply Eq. (2.1).

2.5 $$\begin{aligned} u_1 = \frac{{4{j_4}({j_5}t + {j_4}x)}}{{ - \frac{{3j_4^4\left( { - 3j_2^2 + 3j_5^2 + 2j_2^2{c_1} - 6j_5^2{c_1}} \right) \left( { - 5j_2^2 + j_5^2 + 6j_2^2{c_1} - 2j_5^2{c_1}} \right) }}{{j_2^2{{\left( {j_2^2 + j_5^2} \right) }^2}}} + {{\left( { - \frac{{j_4^2\left( { - 3j_2^2 + 3j_5^2 + 2j_2^2{c_1} - 6j_5^2{c_1}} \right) }}{{{j_2}\left( {j_2^2 + j_5^2} \right) }} + {j_2}t} \right) }^2} + {{\left( {{j_5}t + {j_4}x} \right) }^2}}}. \end{aligned}$$

**Case 2:**

2.6 $$\begin{aligned} {\left\{ \begin{array}{ll} {j_1} = 0,\\ {j_2} = - \frac{{j_4^2(2{c_1} - 3)}}{{{j_3}}},\\ {j_5} = 0,\\ {j_6} = 0,\\ {j_7} = j_3^{{\hspace{1.0pt}} 2},\\ {c_2} = \frac{1}{2}\frac{{j_4^2(2{c_1} - 3)}}{{j_3^2}}. \end{array}\right. } \end{aligned}$$

Using these values, we have solution of the form

2.7 $$\begin{aligned} u_2 = \frac{{4j_4^{{\hspace{1.0pt}} 2}x}}{{j_3^{{\hspace{1.0pt}} 2} + {{\left( {{j_3} - \frac{{j_4^2( - 3 + 2{c_1})t}}{{{j_3}}}} \right) }^2} + j_4^2{x^2}}}. \end{aligned}$$

**Fig. 3:**
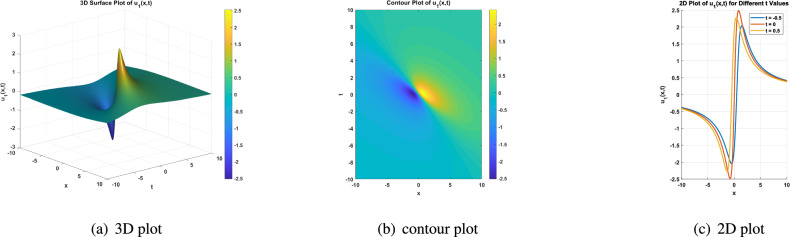
Visualization of 3D, contour, and 2D profiles of LS for Eq. (2.5), when ${j_2} = 1.5,{j_4} = 1.5,{j_5} = 1.5,{c_1} = - 0.05$

**Fig. 4:**
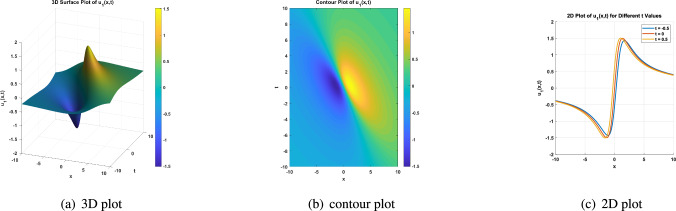
Visualization of 3D, contour, and 2D profiles of LS for Eq. (2.5), when ${j_2} = 1.5,{j_4} = 2.5,{j_5} = 1.5,{c_1} = - 0.05$

**Fig. 5:**
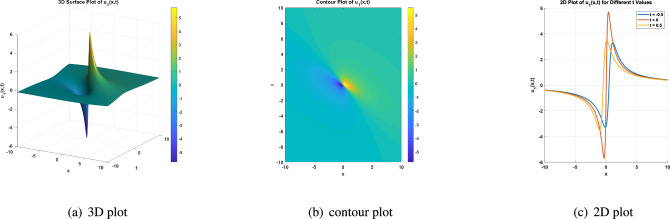
Visualization of 3D, contour, and 2D profiles of LS for Eq. (2.5), when ${j_2} = 1.5,{j_4} = 1.5,{j_5} = 1.5,{c_1} = - 0.01$

## Lump with one kink solution

We examine the subsequent function for a $L_1K$ solution (Rizvi et al. 2025):

3.1 $$\begin{aligned} f = \eta _1^2 + \eta _2^2 + {n_1}{e^{{K_1}}} + {j_7}, \end{aligned}$$

where ${\eta _1} = {j_1}x + {j_2}t + {j_3},$ ${\eta _2} = {j_4}x + {j_5}t + {j_6},$ ${K_1} = {d_1}x + {d_2}t$, and ${j_i}(0 \le i \le 7)$, $d_1$, $d_2$ and $n_1$ are constants. By replacing Eq. (3.1) into Eq. (2.2), we obtain several equations that provide coefficient values by reducing all of the coefficients of the *exp*, *x*, and *t* to zero, as

**Case 1:**

3.2 $$\begin{aligned} {\left\{ \begin{array}{ll} {j_1} = 0,\\ {j_3} = \frac{{3{j_4}{j_5}\left( { - 10{j_4}{j_5}{d_1}{c_1} + 9{j_4}{j_5}{d_1} - 4{j_4}{j_5}{c_3} + j_2^2} \right) }}{{{j_2}{d_1}\left( {j_2^2 - 3j_5^2} \right) }},\\ {j_6} = 0,\\ {j_7} = 0,\\ {d_2} = - \frac{{{d_1}\left( {6{j_4}{j_5}{d_1}{c_1} - 15{j_4}{j_5}{d_1} - 12{j_4}{j_5}{c_3} + j_2^2 + 3j_5^2} \right) }}{{9{j_4}{j_5}}},\\ {c_2} = - \frac{{{d_1}\left( {6{j_4}{j_5}{d_1}{c_1} - 6{j_4}{j_5}{d_1} - 12{j_4}{j_5}{c_3} + j_2^2 + 3j_5^2} \right) }}{{9{j_4}{j_5}}}. \end{array}\right. } \end{aligned}$$

To obtain the $L_1K$ for Eq. (1.3), replace Eq. (3.2) into Eq. (3.1) and apply Eq. (2.1).

3.3 $$\begin{aligned} u_3=\frac{{2\left( {{d_1}{e^\varpi }{m_1} + 2{j_4}\left( {{j_5}t + {j_4}x} \right) } \right) }}{{{e^\varpi }{m_1} + {{\left( {\frac{{3{j_4}{j_5}\left( {j_2^2 + 9{j_4}{j_5}{d_1} - 10{j_4}{j_5}{d_1}{c_1} - 4{j_4}{j_5}{c_3}} \right) }}{{{j_2}\left( {j_2^2 - 3j_5^2} \right) {d_1}}} + {j_2}t} \right) }^2} + {{\left( {{j_5}t + {j_4}x} \right) }^2}}}, \end{aligned}$$

where

$$\begin{aligned} \varpi { = ^{ - \left( {\frac{{{d_1}\left( {j_2^2 + 3j_5^2 - 15{j_4}{j_5}{d_1} + 6{j_4}{j_5}{d_1}{c_1} - 12{j_4}{j_5}{c_3}} \right) t}}{{9{j_4}{j_5}}}} \right) + {d_1}x}}. \end{aligned}$$

**Case 2:**

3.4 $$\begin{aligned} {\left\{ \begin{array}{ll} {j_1} = 0,\\ {j_2} = 0,\\ {j_6} = 0,\\ {j_7} = 0,\\ {d_1} = - \frac{{4{c_3}}}{{10{c_1} - 9}},\\ {d_2} = - \frac{{4{c_3}\left( {48{j_4}{c_1}{c_3} - 56{j_4}{c_3} - 10{j_5}{c_1} + 9{j_5}} \right) }}{{3{j_4}{{\left( {10{c_1} - 9} \right) }^2}}},\\ {c_2} = - \frac{{4{c_3}\left( {48{j_4}{c_1}{c_3} - 44{j_4}{c_3} - 10{j_5}{c_1} + 9{j_5}} \right) }}{{3{j_4}{{\left( {10{c_1} - 9} \right) }^2}}},\\ \end{array}\right. } \end{aligned}$$

Using these values, we have solution of the form

3.5 $$\begin{aligned} u_4=\frac{{2\left( { - \frac{{4{c_3}\exp \left( { - \left( {\frac{{4{c_3}\left( {9{j_5} - 10{j_5}{c_1} - 56{j_4}{c_3} + 48{j_4}{c_1}{c_3}} \right) t}}{{3{j_4}{{\left( { - 9 + 10{c_1}} \right) }^2}}}} \right) - \frac{{4{c_3}x}}{{ - 9 + 10{c_1}}}} \right) {m_1}}}{{ - 9 + 10{c_1}}} + 2{j_1}\left( {{j_3} + {j_1}x} \right) + 2{j_4}\left( {{j_6} + {j_5}t + {j_4}x} \right) } \right) }}{{{j_7} + \exp \left( { - \left( {\frac{{4{c_3}\left( {9{j_5} - 10{j_5}{c_1} - 56{j_4}{c_3} + 48{j_4}{c_1}{c_3}} \right) t}}{{3{j_4}{{\left( { - 9 + 10{c_1}} \right) }^2}}}} \right) - \frac{{4{c_3}x}}{{ - 9 + 10{c_1}}}} \right) {m_1} + {{\left( {{j_3} + {j_1}x} \right) }^2} + {{\left( {{j_6} + {j_5}t + {j_4}x} \right) }^2}}}. \end{aligned}$$

**Fig. 6:**
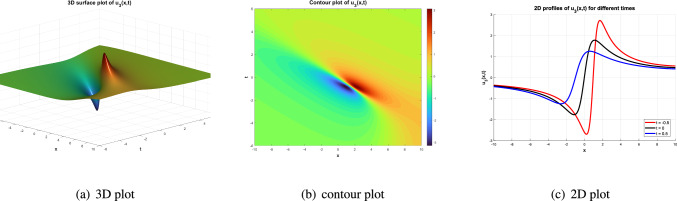
Visualization of 3D, contour, and 2D profiles of $L_1K$ for Eq. (3.3), when ${j_2} = 5,\mathrm{ }{j_4} = 5,\mathrm{ }{j_5} = 10,{d_2} = 0.5,{d_1} = 0.5,{m_1} = 2.5,{c_1} = 1.5,{c_3}\mathrm{ } = - 0.5$

**Fig. 7:**
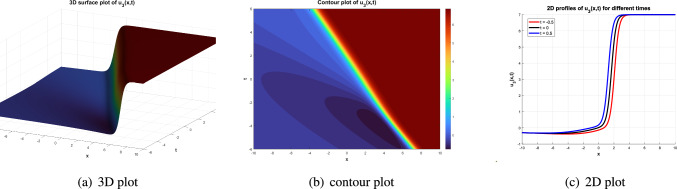
Visualization of 3D, contour, and 2D profiles of $L_1K$ for Eq. (3.3), when ${j_2} = 5,\mathrm{ }{j_4} = 5,\mathrm{ }{j_5} = 10,{d_2} = 0.5,{d_1} = 3.5,{m_1} = 2.5,{c_1} = 1.5,{c_3}\mathrm{ } = - 0.5$

**Fig. 8:**
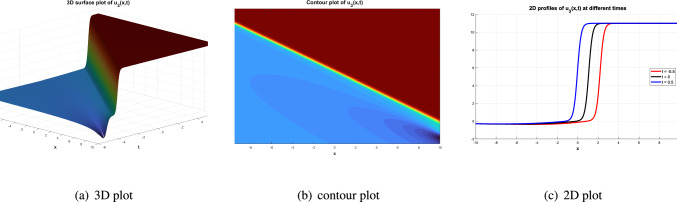
Visualization of 3D, contour, and 2D profiles of $L_1K$ for Eq. (3.3), when ${j_2} = 5,\mathrm{ }{j_4} = 5,\mathrm{ }{j_5} = 10,{d_2} = 0.5,{d_1} = 5.5,{m_1} = 2.5,{c_1} = 1.5,{c_3}\mathrm{ } = - 0.5$

## Lump with two kink solution

We examine the subsequent function for a $L_2K$ solution (Rizvi et al. 2025):

4.1 $$\begin{aligned} f = \eta _1^2 + \eta _2^2 + {m_1}{e^{{K_1}}} + {m_2}{e^{{K_2}}} + {j_7}, \end{aligned}$$

where ${\eta _1} = {j_1}x + {j_2}t + {j_3},$ ${\eta _2} = {j_4}x + {j_5}t + {j_6},$ ${K_1} = {d_1}x + {d_2}t$, ${K_2} = {d_3}x + {d_4}t$, ${j_i}(0 \le i \le 7)$, ${d_i}(0 \le i \le 4)$, $m_1$ and $m_2$ are constants. By replacing Eq. (4.1) into Eq. (2.2), and repeating the same procedure as in $LI_K$, we derive parameter values.

**Case 1:**

4.2 $$\begin{aligned} {\left\{ \begin{array}{ll} {j_1} = 0,\\ \mathrm{ }{j_6} = 0,\\ \mathrm{ }{j_7} = 0,\\ {d_1} = 0,\\ {d_4} = d_3^2 + {c_2},\\ {c_1} = \frac{{11{j_4}d_3^2 - 3{j_4}{c_2} + {j_5}{d_3}}}{{12{j_4}d_3^2}},\\ \mathrm{ }{c_3} = - \frac{{{j_4}d_3^2 - 15{j_4}{c_2} - 13{j_5}{d_3}}}{{24{j_4}{d_3}}}. \end{array}\right. } \end{aligned}$$

To obtain the $L_2K$ for Eq. (1.3), replace Eq. (4.2) into Eq. (4.1) and apply Eq. (2.1).

4.3 $$\begin{aligned} {u_5}(x,t) = \frac{{2({d_3}{e^{(d_3^2 + {c_2})t + {d_3}x}}{m_2} + 2{j_4}({j_5}t + {j_4}x) + 2\sqrt{3} {j_5}({j_3} + {j_2}t + \sqrt{3} {j_5}x))}}{{{e^{{d_2}t}}{m_1} + {e^{(d_3^2 + {c_2})t + {d_3}x}}{m_2} + {{({j_5}t + {j_4}x)}^2} + {{({j_3} + {j_2}t + \sqrt{3} {j_5}x)}^2}}}. \end{aligned}$$

**Case 2:**

4.4 $$\begin{aligned} {\left\{ \begin{array}{ll} {j_1} = 0,\\ {j_6} = 0,\\ {j_7} = 0,\\ {j_2} = \sqrt{3} {j_5},\\ {c_1} = \frac{1}{{12}}\frac{{11{j_4}d_3^2 - 3{j_4}{c_2} + {j_5}{d_3}}}{{{j_4}d_3^2}}. \end{array}\right. } \end{aligned}$$

Using these values, we have solution of the form

4.5 $$\begin{aligned} {u_6}(x,t) = \frac{{2({d_3}{e^{{d_4}t + {d_3}x}}{m_2} + 2{j_4}({j_5}t + {j_4}x))}}{{{e^{{d_2}t}}{m_1} + {e^{{d_4}t + {d_3}x}}{m_2} + {{({j_3} + \sqrt{3} {j_5}t)}^2} + {{({j_5}t + {j_4}x)}^2}}}. \end{aligned}$$

**Fig. 9:**
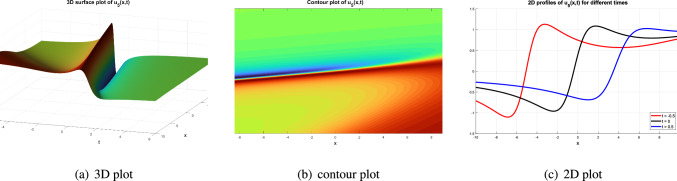
Visualization of 3D, contour, and 2D profiles of $L_2K$ for Eq. (4.3), when ${m_1} = 0.5,\mathrm{ }{m_2} = 5,{j_2} = - 0.05,{j_3} = 0.5,{j_4} = 0.5,{j_5} = - 5,{d_2} = 0.05,{d_3} = 0.5,{c_2} = - 1.5$

**Fig. 10:**
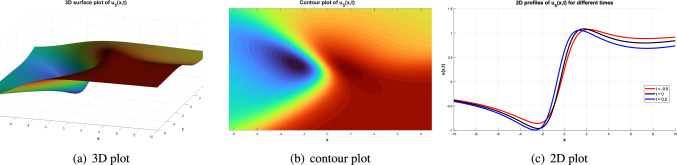
Visualization of 3D, contour, and 2D profiles of $L_2K$ for Eq. (4.3), when ${m_1} = 0.5,\mathrm{ }{m_2} = 0.5,{j_2} = - 0.05,{j_3} = 0.5,{j_4} = 0.5,{j_5} = 0. 5,{d_2} = 0.05,{d_3} = 0.5,{c_2} = - 1.5$

**Fig. 11:**
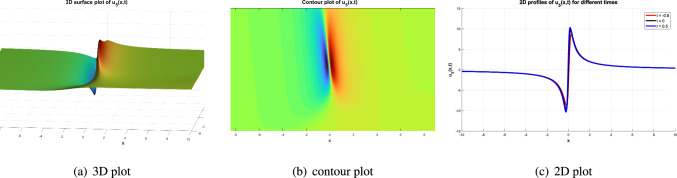
Visualization of 3D, contour, and 2D profiles of $L_2K$ for Eq. (4.3), when ${m_1} = 0.5,\mathrm{ }{m_2} = 0.5,{j_2} = - 0.05,{j_3} = 0.5,{j_4} = 5.5,{j_5} = - 5,{d_2} = 0.05,{d_3} = 0.5,{c_2} = - 1.5$

## Rogue waves solutions

For RWs, we choose *f* as Rizvi et al. (2025):

5.1 $$\begin{aligned} {\left\{ \begin{array}{ll} f(x,t) = \eta _1^2 + \eta _2^2 + {m_1}\cosh (\beta (x,t)) + j_7,\\ {\eta _1} = {j_1}x + {j_2}t + {j_3},\\ {\eta _2} = {j_4}x + {j_5}t + {j_6},\\ \beta (x,t) = {d_1}x + {d_2}t, \end{array}\right. } \end{aligned}$$

where ${j_i}(0 \le i \le 7)$, $d_1, d_2$ are constants. By replacing Eq. (5.1) into Eq. (2.2), we obtain several equations that provide parameter values by reducing all of the coefficients of the *sinh*, *cosh*, *x*, and *t* to zero, as

**Case 1:**

5.2 $$\begin{aligned} {\left\{ \begin{array}{ll} {j_1} = 0,\\ {j_3} = - \frac{{j_4^2\left( {10j_2^2{c_1} + 30j_5^2{c_1} - 9j_2^2 - 27j_5^2} \right) }}{{{j_2}\left( {j_2^2 - 3j_5^2} \right) }},\\ {j_6} = 0,\\ {d_2} = 0,\\ {c_2} = - d_1^2,\\ {c_3}\mathrm{ } = \frac{{3{j_5}\left( {j_2^2 + j_5^2} \right) }}{{4{j_4}\left( {j_2^2 + 3j_5^2} \right) }}. \end{array}\right. } \end{aligned}$$

To obtain the RWs solutions for Eq. (1.3), replace Eq. (5.2) into Eq. (5.1) and apply Eq. (2.1).

5.3 $$\begin{aligned} u_7= \frac{{2(2{j_4}({j_5}t + {j_4}x) + {d_1}{m_1}\sinh ({d_1}x))}}{{{j_7} + {{\left( { - \frac{{j_4^2( - 9j_2^2 - 27j_5^2 + 10j_2^2{c_1} + 30j_5^2{c_1})}}{{{j_2}(g_2^2 - 3j_5^2)}} + {j_2}t} \right) }^2} + {{({j_5}t + {j_4}x)}^2} + {m_1}\cosh ({d_1}x)}}. \end{aligned}$$

**Case 2:**

5.4 $$\begin{aligned} {\left\{ \begin{array}{ll} {j_1} = 0,\\ {j_6} = 0,\\ {d_2} = 0,\\ {j_2} = 0,\\ {j_5} = 4{j_4}{c_3}, \\ {j_7} = - \frac{{j_3^2d_1^2 + j_3^2{c_2} - 10j_4^2{c_1} + 9j_4^2}}{{d_1^2 + {c_2}}}. \end{array}\right. } \end{aligned}$$

Using these values, we have solution of the form

5.5 $$\begin{aligned} {u_8}(x,t) = \frac{{2(2{j_4}(4{j_4}{c_3}t + {j_4}x) + {d_1}{m_1}\sinh ({d_1}x))}}{{j_3^2 + \frac{{ - 9j_4^2 - j_3^2d_1^2 + 10j_4^2{c_1} - j_3^2{c_2}}}{{d_1^2 + {c_2}}} + {{(4{j_4}{c_3}t + {j_4}x)}^2} + {m_1}\cosh ({d_1}x)}}. \end{aligned}$$

**Fig. 12:**
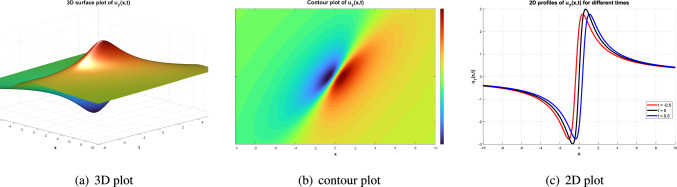
Visualization of 3D, contour, and 2D profiles of RWs for Eq. (5.3), when ${j_2} = - 1.9,{j_4} = 3.5,{j_5} = - 2.5,{j_7} = 5,{m_1} = 0.5,{d_1} = 0.4,{c_1} = 0.9$

**Fig. 13:**
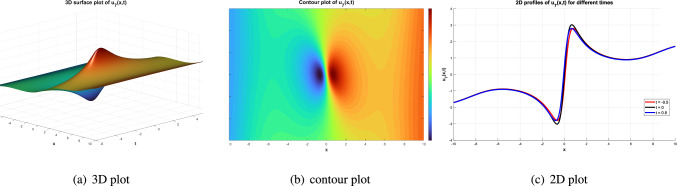
Visualization of 3D, contour, and 2D profiles of RWs for Eq. (5.3), when ${j_2} = - 1.9,{j_4} = 3.5,{j_5} = - 2.5,{j_7} = 5,{m_1} = 0.5,{d_1} = 1,{c_1} = 0.9$

**Fig. 14:**
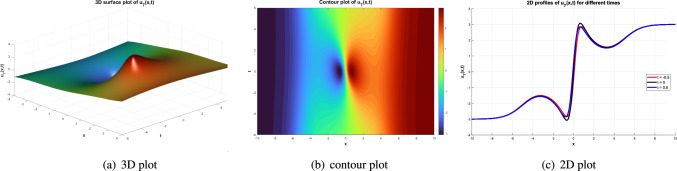
Visualization of 3D, contour, and 2D profiles of RWs for Eq. (5.3), when ${j_2} = - 1.9,{j_4} = 3.5,{j_5} = - 2.5,{j_7} = 5,{m_1} = 0.5,{d_1} = 1.5,{c_1} = 0.9$

## Breathers wave solutions

For BWs, we choose *f* as Rizvi et al. (2025):

6.1 $$\begin{aligned} f = {e^{ - q\eta }} + {m_1}{e^{q\eta }} + {m_2}\cos \left( {{q_1}{\eta _2}} \right) + {j_6}, \end{aligned}$$

where ${\eta _1} = {j_1}x + {j_2}t + {j_3}$, ${\eta _2} = {j_4}x + {j_5}t,$ ${j_i}(0 \le i \le 7)$, $m_1, m_2$ are constants. By replacing Eq. (6.1) into Eq. (2.2), and repeating the same procedure as in $LI_K$, we derive parameter values.

**Case 1:**

6.2 $$\begin{aligned} {\left\{ \begin{array}{ll} {j_2} = 0,\\ {j_5} = - \frac{{{j_4}\left( {10j_1^2{c_1}{q^2} - 2j_4^2{c_1}q_1^2 - 9j_1^2{q^2} + 3j_4^2q_1^2 - 12{j_1}{c_3}q} \right) }}{{3{j_1}q}},\\ {j_6} = 0,\\ {c_2} = \frac{2}{3}{c_1}\left( {j_1^2{q^2} + j_4^2q_1^2} \right) . \end{array}\right. } \end{aligned}$$

To obtain the BWs solutions for Eq. (1.3), replace Eq. (6.2) into Eq. (6.1) and apply Eq. (2.1).

6.3 $$\begin{aligned} {u_9} = \frac{{2( - {j_1}q{e^{ - q\left( {{j_3} + {j_1}x} \right) }} + {j_1}{m_1}q{e^{q\left( {{j_3} + {j_1}x} \right) }} - {j_4}{m_2}{q_1}\sin \left( {{q_1}\varpi } \right) )}}{{{e^{ - q\left( {{j_3} + {j_1}x} \right) }} + {m_1}{e^{q\left( {{j_3} + {j_1}x} \right) }} + {m_2}\cos \left( {{q_1}\varpi } \right) }}, \end{aligned}$$

where

$$\begin{aligned} \varpi = ( - \frac{{{j_4}\left( { - 12{j_1}{c_3}q - 9j_1^2{q^2} + 10j_1^2{c_1}{q^2} + 3j_4^2q_1^2 - 2j_4^2{c_1}q_1^2} \right) t}}{{3{j_1}q}} + {j_4}x). \end{aligned}$$

**Case 2:**

6.4 $$\begin{aligned} {\left\{ \begin{array}{ll} {j_2} = 0,{j_6} = 0,{c_2} = \frac{{4j_1^2{j_4}{c_1}{q^2} - 3j_1^2{j_4}{q^2} + j_4^3q_1^2 - 4{j_1}{j_4}{c_3}q + {j_1}{j_5}q}}{{{j_4}}},{m_1} = 0. \end{array}\right. } \end{aligned}$$

Using these values, we have solution of the form

6.5 $$\begin{aligned} {u_{10}} = \frac{{2\left( { - {j_1}q{e^{ - q\left( {{j_3} + {j_1}x} \right) }} - {j_4}{m_2}{q_1}\sin \left( {{q_1}\left( {{j_5}t + {j_4}x} \right) } \right) } \right) }}{{{e^{ - q\left( {{j_3} + {j_1}x} \right) }} + {m_2}\cos \left( {{q_1}\left( {{j_5}t + {j_4}x} \right) } \right) }}. \end{aligned}$$

**Fig. 15:**
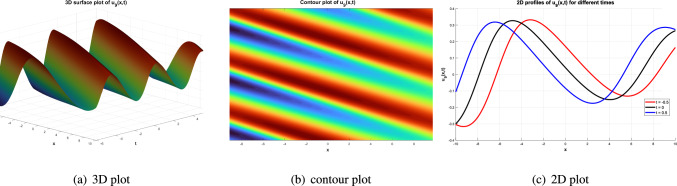
Visualization of 3D, contour, and 2D profiles of BWs for Eq. (6.3), when $q = - 0.2,{m_1} = 0.7,{m_2} = 0.6,{q_1} = 0.8,{j_1} = 0.3,{j_3} = 1.5,{j_4} = 0.5,{c_1} = 2,{c_3} = 1$

**Fig. 16:**
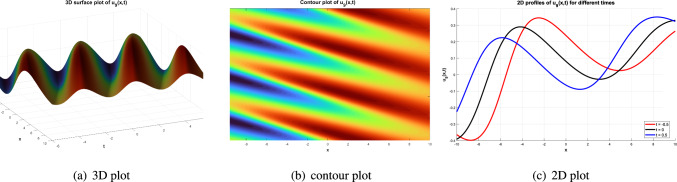
Visualization of 3D, contour, and 2D profiles of BWs for Eq. (6.3), when $q = - 0.2,{m_1} = 0.7,{m_2} = 0.6,{q_1} = 0.8,{j_1} = 0.6,{j_3} = 1.5,{j_4} = 0.5,{c_1} = 2,{c_3} = 1$

## Periodic wave solution

For PWs, we choose *f* as Rizvi et al. (2025):

7.1 $$\begin{aligned} {\left\{ \begin{array}{ll} f(x,t) = \eta _1^2 + \eta _2^2 + {m_1}\cos \beta + {j_7},\\ {\eta _1} = {j_1}x + {j_2}t + {j_3},\\ {\eta _2} = {j_4}x + {j_5}t + {j_6},\\ \beta = {d_1}x + {d_2}t. \end{array}\right. } \end{aligned}$$

where ${j_i}$, $1 \le i \le 7$, ${d_1}$, ${d_2}$, and ${m_1}$ are constants. By inserting Eq. (7.1) into Eq. (2.2), we obtain several equations that provide parameter values by reducing all of the coefficients of the *sin*, *cos*, *x*, and *t* to zero, as

**Case 1:**

7.2 $$\begin{aligned} {\left\{ \begin{array}{ll} {j_2} = \frac{{j_5^2}}{{4{j_1}{c_3}}},\\ {j_2} = \sqrt{\frac{{{c_2}}}{{{c_1} - 3}}},\\ {j_3} = 0,\\ {j_4} = 0,\\ {j_7} = 0,\\ \end{array}\right. } \end{aligned}$$

To obtain the BWs solutions for Eq. (1.3), replace Eq. (7.2) into Eq. (7.1) and apply Eq. (2.1).

7.3 $$\begin{aligned} {u_{11}} = \frac{{2(2{j_1}(\frac{{j_5^2t}}{{4{j_1}{c_3}}} + {j_1}x) - \sqrt{\frac{{{c_2}}}{{ - 3 + {c_1}}}} {m_1}\sin \left( {{d_2}t + \sqrt{\frac{{{c_2}}}{{ - 3 + {c_1}}}} x} \right) )}}{{{{({j_6} + {j_5}t)}^2} + {{(\frac{{j_5^2t}}{{4{j_1}{c_3}}} + {j_1}x)}^2} + {m_1}\cos \left( {{d_2}t + \sqrt{\frac{{{c_2}}}{{ - 3 + {c_1}}}} x} \right) }}. \end{aligned}$$

**Case 2:**

7.4 $$\begin{aligned} {\left\{ \begin{array}{ll} {j_2} = 0,{j_6} = 0,{c_2} = \frac{{4j_1^2{j_4}{c_1}{q^2} - 3j_1^2{j_4}{q^2} + j_4^3q_1^2 - 4{j_1}{j_4}{c_3}q + {j_1}{j_5}q}}{{{j_4}}},{m_1} = 0. \end{array}\right. } \end{aligned}$$

Using these values, we have solution of the form

7.5 $$\begin{aligned} {u_{12}} = \frac{{2(2{j_1}({j_2}t + {j_1}x) - \frac{{2{c_2}{m_1}\sin \left( {\frac{{4j_1^2( - 3 + 2{c_1}){c_3}t}}{{j_6^2{c_2}}} + \frac{{2{c_2}\sqrt{x} }}{{ - 3 + 2{c_1}}}} \right) }}{{ - 3 + 2{c_1}}})}}{{j_6^2 + {{({j_2}t + {j_1}x)}^2} + {m_1}\cos \left( {\frac{{4j_1^2( - 3 + 2{c_1}){c_3}t}}{{j_6^2{c_2}}} + \frac{{2{c_2}\sqrt{x} }}{{ - 3 + 2{c_1}}}} \right) }}. \end{aligned}$$

**Fig. 17:**
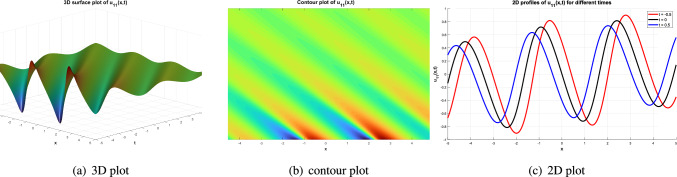
Visualization of 3D, contour, and 2D profiles of PWs for Eq. (7.3), when ${j_1} = 0.5,{j_5} = 0.5,{j_6} = 5,{d_2} = 1.5,{m_1} = 5,{c_1} = 5,{c_2} = 7,{c_3} = 1.5$

**Fig. 18:**
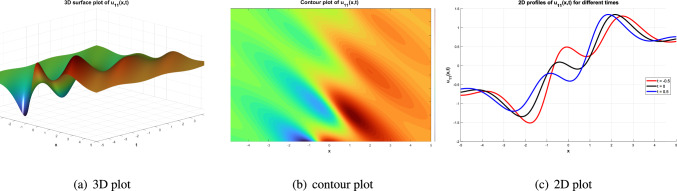
Visualization of 3D, contour, and 2D profiles of PWs for Eq. (7.3), when ${j_1} = 2.5,{j_5} = 0.5,{j_6} = 5,{d_2} = 1.5,{m_1} = 5,{c_1} = 5,{c_2} = 7,{c_3} = 1.5$

**Fig. 19:**
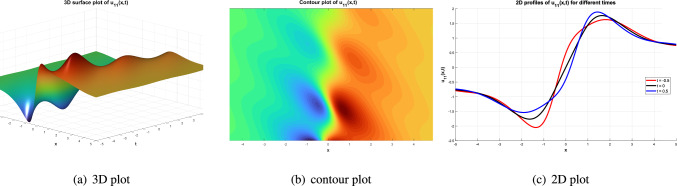
Visualization of 3D, contour, and 2D profiles of PWs for Eq. (7.3), when ${j_1} = 4.5,{j_5} = 0.5,{j_6} = 5,{d_2} = 1.5,{m_1} = 5,{c_1} = 5,{c_2} = 7,{c_3} = 1.5$

## Multi-wave solution

For MWs, we choose (Rizvi et al. 2025).

8.1 $$\begin{aligned} f = {d_0}\cosh \left( {{j_1}\eta + {j_2}} \right) + {d_1}\cos \left( {{j_3}\eta + {j_4}} \right) + {d_2}\cosh \left( {{j_5}\eta + {j_6}} \right) , \end{aligned}$$

where ${j_i},1 \le i \le 7$, and ${d_i},0 \le i \le 2$ are real parameters. By replacing Eq. (8.1) into Eq. (2.2), we obtain several equations that provide parameter values by reducing all of the coefficients of the *sinh*, *cosh* functions to zero, as

**Case 1:**

8.2 $$\begin{aligned} {\left\{ \begin{array}{ll} {j_1} = - {j_7},\\ {j_3} = 0,\\ {j_6} = 0,\\ {c_1} = \frac{{3j_4^2 - j_7^2 + 2{c_2}}}{{2({j_4} - {j_7})({j_4} + {j_7})}}. \end{array}\right. } \end{aligned}$$

To obtain the MWs solutions for Eq. (1.3), replace Eq. (8.2) into Eq. (8.1) and apply Eq. (2.1).

8.3 $$\begin{aligned} {u_{13}} = \frac{{2( - {j_4}{f_1}\sin \left( {{j_5}t + {j_4}x} \right) - {j_7}{f_0}\sinh \left( {{j_2}t - {j_7}x} \right) + {j_7}{f_2}\sinh \left( {{j_9} + {j_8}t + {j_7}x} \right) )}}{{{j_{10}} + {f_1}\cos \left( {{j_5}t + {j_4}x} \right) + {f_0}\cosh \left( {{j_2}t - {j_7}x} \right) + {f_2}\cosh \left( {{j_9} + {j_8}t + {j_7}x} \right) }}. \end{aligned}$$

**Fig. 20:**
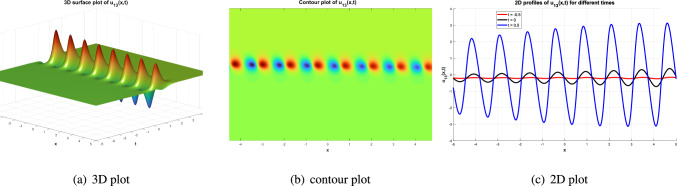
Visualization of 3D, contour, and 2D profiles of MWs for Eq. (8.3), when ${j_2} = - 2.5,{j_4} = 5,{j_5} = 0.5,{j_7} = 0.1,{j_8} = 5,{j_9} = - 4,{j_{10}} = 0.1,{f_0} = 0.4,{f_1} = 0.7,{f_2} = 0.7$

**Fig. 21:**
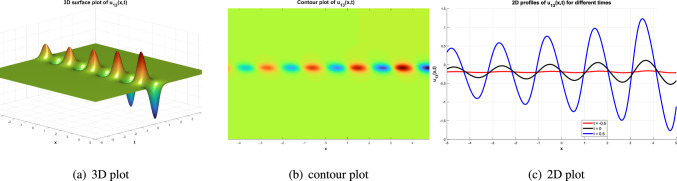
Visualization of 3D, contour, and 2D profiles of MWs for Eq. (8.3), when ${j_2} = - 2.5,{j_4} = 3,{j_5} = 0.5,{j_7} = 0.1,{j_8} = 5,{j_9} = - 4,{j_{10}} = 0.1,{f_0} = 0.4,{f_1} = 0.7,{f_2} = 0.7$

**Fig. 22:**
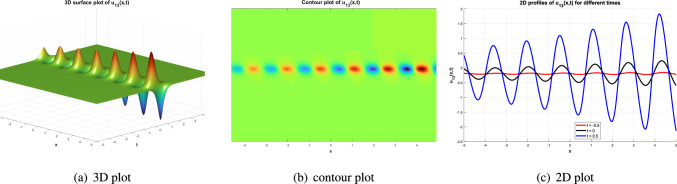
Visualization of 3D, contour, and 2D profiles of MWs for Eq. (8.3), when ${j_2} = - 2.5,{j_4} = 4,{j_5} = 0.5,{j_7} = 0.1,{j_8} = 5,{j_9} = - 4,{j_{10}} = 0.1,{f_0} = 0.4,{f_1} = 0.7,{f_2} = 0.7$

## Results and discussion

Soliton structures play a significant role in describing wave propagation in complex physical and biological systems. Exact analytical techniques have been widely used to obtain stable traveling and optical soliton solutions for several nonlinear evolution equations, demonstrating their importance in modeling pulse transmission, nonlinear interactions, and energy localization (Ullah et al. 2025; Bilal et al. 2024). Motivated by these developments, the present section provides a detailed physical interpretation of the obtained wave solutions and examines the influence of different parameters on the dynamics of nonlinear waves in arterial blood flow. In this section, we present a more detailed physical meaning of the wave solutions obtained and consider the effect of different parameters on the dynamics of nonlinear waves in arteries. Figure 1 illustrates the schematic representation of blood vessels and the associated hemodynamic flow behavior. Figure 2 presents a conceptual visualization of nonlinear wave structures and their qualitative role in blood flow dynamics. Figures 3, 4, and 5 describe LS for the parameters values $${j_2} = 1.5,{j_4} = 1.5,{j_5} = 1.5,{c_1} = - 0.05,$$ $${j_2} = 1.5,{j_4} = 2.5,{j_5} = 1.5,{c_1} = - 0.05, \mathrm{and} {j_2} = 1.5,$$ $${j_4} = 1.5,{j_5} = 1.5,{c_1} = - 0.01$$. These plots indicate that as the value of $$c_1$$ decreases, both the peak amplitude and magnitude of solution increase at each level so that the LS have decreasing spatial widths. Thus, as $$c_1$$ decreases, the amount of heterogeneity will produce greater concentrated pressure or fluid pulses in the vessel system. On a physiological level, this means that when $$c_1$$ is lower, the rigidity or irregularity of the vessel wall may increase resulting in a greater concentration of localized disturbances that could be potentially damaging. Figures 6, 7, and 8 describe $$L_1K$$ for the parameters values $${j_2} = 5,\mathrm{ }{j_4} = 5,\mathrm{ }{j_5} = 10,{d_2} = 0.5,$$$${d_1} = 0.5,{m_1} = 2.5,{c_1} = 1.5,{c_3}\mathrm{ } = - 0.5, {j_2} = 5,\mathrm{ }{j_4} = 5,$$ $$\mathrm{ }{j_5} = 10,{d_2} = 0.5, {d_1} = 3.5,{m_1} = 2.5,{c_1} = 1.5,{c_3}\mathrm{ } = - 0.5,$$$$\: \mathrm{and} \:{j_2} = 5, \mathrm{ }{j_4} = 5,\mathrm{ }{j_5} = 10,{d_2} = 0.5, {d_1} = 5.5,$$ $${m_1} = 2.5,{c_1} = 1.5,{c_3}\mathrm{ } = - 0.5$$. The visualizations indicate that increasing $$d_1$$ increases the strength of the kink component resulting in steeper transitions and larger differences between the positive and negative sides of $$L_1K$$ solution. These parameters may therefore be interpreted to provide a means of demonstrating very large changes in vessel characteristic properties or those caused by external forces. Extremely high values of $$d_1$$ may be associated with sudden changes in the geometry of the vessel or the stiffness of the vessel, and they might also initiate extreme pressure events. Figures 9, 10, and 11 describe $$L_2K$$ for the parameters values $${m_1} = 0.5,\mathrm{ }{m_2} = 5,{j_2} = - 0.05,{j_3} = 0.5,{j_4} = 0.5,$$$${j_5} = - 5,{d_2} = 0.05,{d_3} = 0.5,{c_2} = - 1.5, {m_1} = 0.5,$$ $$\mathrm{ }{m_2} = 0.5,{j_2} = - 0.05,{j_3} = 0.5,{j_4} = 0.5,{j_5} = - 5,$$$${d_2} = 0.05,{d_3} = 0.5,{c_2} = - 1.5, \mathrm{and}\: {m_1} = 0.5, $$ $$\mathrm{ }{m_2} = 0.5,{j_2} = - 0.05,{j_3} = 0.5,{j_4} = 5.5, {j_5} = - 5,$$$${d_2} = 0.05,{d_3} = 0.5,{c_2} = - 1.5$$. We can observe, by increasing $$j_4$$ reduces the peak amplitude of wave describing enhanced diffusion along the vessel length. Figures 12, 13, and 14 describe RWs for the parameters values $${j_2} = - 1.9,{j_4} = 3.5,{j_5} = - 2.5,{j_7} = 5,$$$${m_1} = 0.5,{d_1} = 0.4,{c_1} = 0.9,{j_2} = - 1.9,{j_4} = 3.5,$$ $${j_5} = - 2.5,{j_7} = 5,{m_1} = 0.5,{d_1} = 1,{c_1} = 0.9\: \text {and}\:$$ $${j_2} = - 1.9,{j_4} = 3.5,{j_5} = - 2.5,{j_7} = 5,{m_1} = 0.5,$$$${d_1} = 1.5,{c_1} = 0.9$$. These figures describe increasing $$d_1$$ sharper the RWs peaks and stronger energy localization. Figures 15, and 16 describe BWs for the parameters values $$q = - 0.2,{m_1} = 0.7,{m_2} = 0.6,{q_1} = 0.8,{j_1} = 0.3,$$$${j_3} = 1.5,{j_4} = 0.5,{c_1} = 2,{c_3} = 1, \mathrm{and} q = - 0.2,{m_1} = 0.7,$$ $${m_2} = 0.6,{q_1} = 0.8,{j_1} = 0.6,{j_3} = 1.5,{j_4} = 0.5,$$$${c_1} = 2,{c_3} = 1$$. Figures 17, 18, and 19 describe PWs for the parameters values $${j_1} = 0.5,{j_5} = 0.5,{j_6} = 5,{d_2} = 1.5,{m_1} = 5,$$$${c_1} = 5,{c_2} = 7,{c_3} = 1.5,{j_1} = 2.5,{j_5} = 0.5,{j_6} = 5,$$ $${d_2} = 1.5,{m_1} = 5,{c_2} = 7,{c_3} = 1.5 ,\: \mathrm{and} \: {j_1} = 4.5,$$ $${j_5} = 0.5,{j_6} = 5,{d_2} = 1.5,{m_1} = 5,{c_1} = 5,{c_2} = 7,{c_3} = 1.5.$$ As $$j_1$$ increases, we see higher frequency oscillations at the same bounded amplitude level. This characteristic is consistent with the physiological phenomenon that increases in heart rate correspond to more frequent oscillations in pressure. Figures 20, 21, and 22 describe MWs for the parameters values $${j_2} = - 2.5,{j_4} = 5,{j_5} = 0.5,{j_7} = 0.1,{j_8} = 5,{j_9} = - 4,$$$${j_{10}} = 0.1,{f_0} = 0.4,{f_1} = 0.7,{f_2} = 0.7, {j_2} = - 2.5,$$ $${j_4} = 3,{j_5} = 0.5,{j_7} = 0.1,{j_8} = 5,{j_9} = - 4,{j_{10}} = 0.1,$$$${f_0} = 0.4,{f_1} = 0.7,{f_2} = 0.7,\: \mathrm{and} \:{j_2} = - 2.5,{j_4} = 4,$$ $${j_5} = 0.5,{j_7} = 0.1,{j_8} = 5,{j_9} = - 4,{j_{10}} = 0.1,$$$${f_0} = 0.4,{f_1} = 0.7,{f_2} = 0.7.$$ These figures illustrate that $$j_7$$, $$j_8$$ and $$j_9$$ describe how hyperbolic and trigonometric waves interact and how these new interactions are being created. Altering $$j_7$$, $$j_8$$ or $$j_9$$ by even small amounts can substantially change the interference pattern, creating either constructive or destructive interactions. The fact that these parameters are so sensitive to change confirms the non-linear characteristics of wave propagation through complex vascular networks. In general, the graphical analysis supports the notion that the nonlinear Murray equation is highly affected by the changing values of various parameters. The parameters responsible for heterogeneity and non-linearity primarily determine the magnitude and location of the waves, while the parameters of diffusion and frequency control the width and oscillatory nature of the waves. These results emphasize the need to incorporate realistic properties of the vessel into mathematical models of blood flow in order to provide accurate representations of both normal and abnormal hemodynamic behaviour.

## Conclusions

In order to identify new soliton solutions in mathematical biology, this study offers a thorough investigation of the LS, $L_1K$, $L_1K$, RWs, BWs, PWs, and MWs solutions of the nonlinear Murray equation using the proper transformations. We used graphs that included 3D, contour and 2D plots with a few unique elements to show how these solutions behaved. The research demonstrates how the wave parameters control the wave’s amplitude, velocity, and singularity, making it simpler to regulate these properties. The mathematical insights gained from this work will have significant biomedical applications. The LS provide a mathematical analogy for localized pressure pulses and RWs illustrate extreme spikes in arterial surges. The BWs describe the pulsatile, repetitive nature of the flow pattern. MWs solutions illustrate the dynamic nature of arterial networks. The study highlight importance of nonlinear theory in both the normal and pathological physiology of the circulatory system. To the best of our knowledge, this is the first comprehensive study that reports a broad range of wave solutions for the nonlinear Murray equation using the proposed ansatz transformations. The future research can be devoted to the applications of the suggested technique in solving high-dimensional nonlinear problems and some other physically interesting equations associated with the field of fluid mechanics and biology. Further research can also be done to examine the effects of considering fractional order derivatives. Finally, the stability and bifurcation analysis of the obtained results need to be analyzed.

## Data Availability

No datasets were generated or analysed during the current study.
